# Cerium oxide nanoparticles embedded thin-film nanocomposite nanofiltration membrane for water treatment

**DOI:** 10.1038/s41598-018-23188-7

**Published:** 2018-03-21

**Authors:** Sonia R. Lakhotia, Mausumi Mukhopadhyay, Premlata Kumari

**Affiliations:** 1 Applied Chemistry Department, Sardar Vallabhbhai National Institute of Technology, Surat, 395007 Gujarat India; 2 Department of Chemical Engineering, Sardar Vallabhbhai National Institute of Technology, Surat, 395007 Gujarat India

## Abstract

In this paper, a new approach to synthesize thin-film nanocomposite membranes using cerium oxide (CeO_2_) nanoparticles (NPs) by pre-seeding interfacial polymerization method was reported. Prepared membranes were examined using contact angle, molecular weight cut-off (MWCO), scanning electron microscopy (SEM), Fourier transform infrared spectroscopy (FTIR) and scanning probe microscopy (SPM) to observe its hydrophilicity, pore size, morphology, surface chemistry, and roughness, respectively. Surface charges of the prepared membranes were also qualitatively calculated with the help of contact angle measurements by using the Grahame equation. MWCO studies revealed >90% polyethylene glycol (M.W. 1500 Da) rejection, which was fitted in the range of nanofiltration. By increasing the concentration of CeO_2_ NPs, flux (33.12 to 41.28 L/m^2^h), hydrophilicity (77.3 to 51.1°) and surface charges (−7.58 to −13.39 mC/m^2^) of the membranes was successfully improved, and also showed the high (>90%) salt rejections. The CeO_2_ embedded membrane was also found out in successful prevention from the attack of bacteria (*Escherichia coli*) compared to pure polyamide (PA) membrane and confirmed through SEM and viable cell count method. The membrane performances were also evaluated using seawater for fouling study and found that CeO_2_ embedded surface increased the rejection of hydrophobic contaminants, and notably reduced the fouling.

## Introduction

The scarcity of clean water along with the increasing contamination of river bodies is one of the most severe problems faced worldwide^1,2^. Conventional water filtration technique is energy consuming processes and also fails to fulfill all the requirements of human beings. Thus developing effective water treatment approaches is one of the most indispensable needs of the hour^3^. Nowadays membrane separation technique has gained a significant role in separation and purification of water as they are eco-friendly and faster. Nanofiltration (NF) membrane is one of the largely used separation technique^4,5^ especially in desalination and various industrial applications^6^ for the production of clean and safe water. However, polymeric membranes are not efficient due to its low stability with limited life durability, selectivity, and hydrophobic nature^7–9^. Hydrophobic nature of membrane causes severe fouling especially bio-fouling causing a decline in the separation efficiency and flux in water treatment. Several methods have been considered in order to reduce fouling^6^. Therefore at the present time, nanocomposite membrane prepared by embedding nanomaterials is gaining a great importance in the field of membrane separation for water treatment^10^. The recent study has been focused on low-cost, durable anti-fouling nanocomposite membrane for the water treatment. These nanoparticles (NPs) embedded membranes are control over polymeric membrane^11^ as it can be easily tailored as per the conditions of separation with improved smoothness, stability, hydrophilicity, and antibacterial activity^12,13^.

In past years, various NPs like TiO_2_^6,14,15^, zeolite^16–18^, and silica^5,19^ have been used for the preparation of nanocomposite membrane which shows enhanced flux, fouling-resistant, and salt rejection properties. But comparing with different types of fouling, bio-fouling is a bigger problem in the application of NF membrane^2,15^ in which bacteria grow on the membrane surface during the separation process. Due to their self-reproducing nature, it is very difficult to maintain the efficiency of membrane separation. Silver^20–22^, carbon nanotubes (CNTs)^20,23^ and graphene oxide (GO)^24^ NPs are the most considered nanofillers source that acts as an anti-bacterial agent to prevent bio-fouling from the membrane. Even though these NPs are very effective, but are expensive and not easily available. Hence exploration of such material that has anti-biofouling properties is very necessary. Copper (Cu) NPs is to be considered as an antibacterial material against various microorganisms including viruses, bacteria, and fungi^25^. But, its major drawback is its stability and can be easily aggregated, which can decrease its antibacterial activity.

In particular, cerium oxide (CeO_2_) NPs have found great potential applications as nanofiller due to its low cost, high surface area and quick transformation between Ce^+3^ ↔ Ce^+4^ which enhance its antioxidant properties^26–28^. CeO_2_ NPs is an effective biocide against various bacterial strains^26^ and used in many known applications such as catalysis, sensors, purifying contaminated water and also in various industrial applications^27^. Still, only limited work has been reported and available on antibacterial activity in water treatment process with CeO_2_ NPs. Pelletier *et al*. and Thill *et al*. have been reported antimicrobial activity of CeO_2_ NPs against *Escherichia coli (E. coli)*^29,30^, whereas Fang *et al*. have shown the antibacterial activity against *Nitrosomonas europaea*^31^. The colony forming units (CFU) after contact with CeO_2_ NPs is lethal to bacteria. Peng *et al*. and his co-workers have been used CeO_2_ supported CNTs composite adsorbent to study the adsorption capacity of arsenate from the potable water^32^. Authors observed that the prepared composite material (CeO_2_-CNTs) have a great potential in removing arsenate efficiently compared to only CNTs due to its high electrostatic attraction between CeO_2_-CNTs and arsenic (V) anions. The electrostatic attraction can facilitate and improve the adsorption of arsenic (V)^32^. Composites of CeO_2_ NPs on CNTs are also used to adsorb the chromium (VI) ion from drinking water^33^. Therefore, CeO_2_ NPs are expected to be suitable NPs as filler in the preparation of nanocomposite membrane.

There are two methods for preparing nanocomposite membrane: (i) coating and (ii) blending. The coating of NPs like TiO_2_, zeolite, silica, CNTs, silver, GO has been widely used and is an easy way to modify the membranes^34–36^. The literature studies show that coating method involves a thin-film layer formation with NPs on a porous support without a major change in the membrane structure and morphology. Polysulfone (PS)^3^ and polyethersulfone (PES)^2^ have been commonly used as a porous support in the water filtration process because they have high strength and tunable pore size chemistry. Rahimpour *et al*. have discussed the comparison between membrane preparation by depositing and blending of NPs method. Authors found that the coating of NPs provides high surface layer sustainability by generating stronger physical/chemical bonds as compared to blending and also effectively improve the membrane performance^10^ and it’s anti-fouling ability.

By considering the advantages of CeO_2_ NPs, in this work, CeO_2_ NPs embedded thin-film nanocomposite (TFN) membranes are prepared on PES porous support by pre-seeding interfacial polymerization method and analyzed by molecular weight cut-off (MWCO), contact angle, scanning electron microscopy (SEM), attenuated total reflection (ATR) attached with Fourier transform infrared (FTIR) spectroscopy, transmission electron microscopy (TEM) and scanning probe microscopy (SPM). The TFN CeO_2_ membrane performance is also evaluated using a cross-flow filtration setup for rejection performance (with different salt solution) and anti-fouling study using saltwater (seawater) collected from Suvali beach, Surat, India. In addition to this work, an antibacterial activity of this TFN membrane was also checked with *E. coli*. strain.

## Results and Discussion

### Membrane preparation

**Figure 1:**
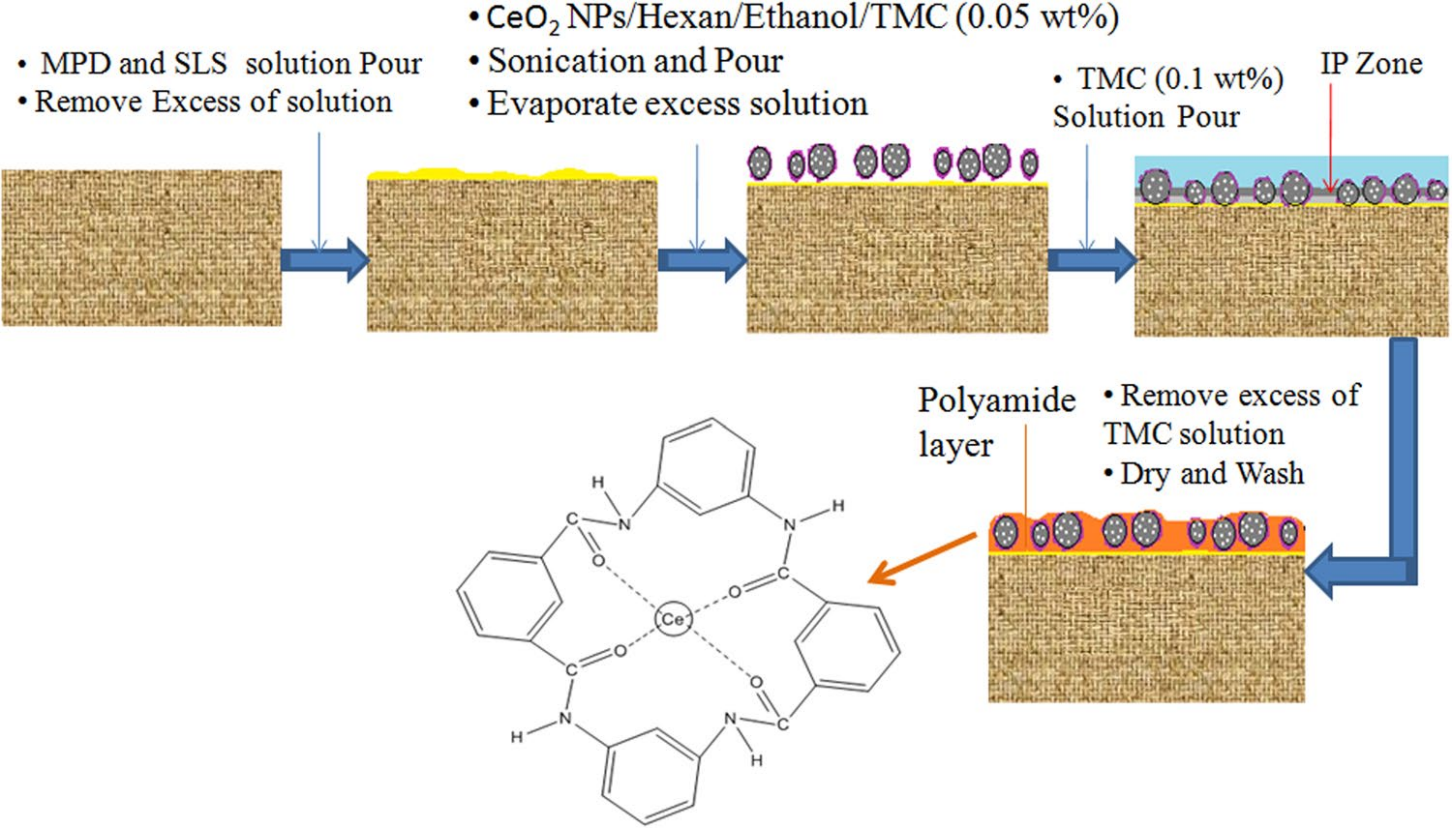
Steps of pre-seeding interfacial polymerization method for CeO_2_ NPs embedded TFN membrane.

Selection of an appropriate method for incorporating the particular NPs onto the membrane surface is as important as that of choosing the right NPs. There are two methods for embedding NPs into the polyamide (PA) thin-film membrane viz. conventional and pre-seeding polymerization^37,38^. Conventional methods are of two types depending on, whether the NPs are dispersed in m-phenylenediamine (MPD) or trimesoyl chloride (TMC) solution. If NPs are dispersed in TMC, an interfacial polymerization (IP) zone is formed below the NPs. Since the NPs are on the top most layers of the membrane it gets agglomerated and also has a great chance of leaching. If NPs are dispersed in MPD solution, IP zone is formed above the NPs followed by the formation of PA layer. Since the PA layer is on the top, fouling will be more^37^. To overcome this problem, an intermediate pre-seeding hexane solution containing a low concentration of TMC, ethanol, and NPs is introduced between MPD and TMC solution to assist the dispersion of NPs on the top surface of the membrane support. This resulted in the formation of an IP zone at the same layer (neither above nor below the NPs) of the membrane where the NPs are present (Fig. S1). Here, the PA layer is formed with the embedded NPs. Since a good bonding between the NPs and PA layer exists, problems like agglomeration and leaching of the NPs, fouling etc. are rectified. Thus to prepare self-cleaning and defect-free TFN NF membrane, pre-seeding interfacial polymerization by ultrasonication and stirring method is used to promote the proper dispersion of CeO_2_ NPs within the PA layer (Fig. 1).

In this method, CeO_2_ NPs (X-ray diffraction spectra of the available CeO_2_ NPs shown in Fig. S2) hybrid NF membranes were prepared as 0-M1, 0.05-M2, 0.1-M3, 0.2-M4 and 0.3-M5. First, the aqueous liquid of sodium lauryl sulfate 0.15% (w/v) and MPD 2% (w/v) is slowly poured onto the PES support. After approximately 3 min, MPD dipped membranes are put on a sheet and moved with a roller to remove excess MPD solution. Then, a particular amount of CeO_2_ NPs are dispersed in an organic phase of hexane, TMC and ethanol solution, separately, and subjected to sonication and stirring, where CeO_2_ NPs are coordinately attached with the carbonyl group of TMC to form well dispersed pre-assembled CeO_2_ NPs. CeO_2_ NPs present in the solution (w/v) are 0% (0-M1), 0.05% (0.05-M2), 0.1% (0.1-M3), 0.2% (0.2-M4) and 0.3% (0.3-M5) respectively.This well dispersed sonicated solution is spread on MPD coated PES support membrane. This pre-assembled CeO_2_ NPs forms IP zone at the same surface where the CeO_2_ NPs are present and formed the PA layer with the CeO_2_ NPs. After the evaporation of excess solution, 0.1% (w/v) organic solution of TMC is immediately poured, followed by 1 min polymerization reaction and then, rinsed with pure water and characterized by further study.

### Surface study and pore size

Porous PES support can be covered with an increasing concentration of CeO_2_ NPs, which increases the hydrophilicity, surface charge and MWCO of the CeO_2_-PA TFN membrane and the results are shown in Table 1. More spreading of water, lesser the angle and the surface is more hydrophilic. The increase in hydrophilicity is due to the formation of nanovoids around the interface of CeO_2_ NPs and PA layer, which directly affects the water uptake and diffusivity of the CeO_2_-PA TFN membrane. The surface charge of the PA layer coated (0-M1) membrane is −7.58 mC/m^2^. That is closer to the results of other researchers, where the value is −7.2 mC/m^2^ at 7.0 pH^4^. With increasing CeO_2_ NPs concentration, the surface charge improves up to −13.39 mC/m^2^. Highly negatively charged CeO_2_-PA TFN membrane is not attracted the natural foulants (negatively charged substances), thus shows its increased anti-fouling ability impart by embedded CeO_2_ NPs. Moreover, MWCO of the prepared CeO_2_-PA TFN membranes is studied by the rejection performance of  polyethylene glycol (PEG) with different molecular weights (600, 1000, 1500 Da) (Table S1). Prepared CeO_2_-PA TFN membranes show around 90% rejections with PEG 1500 Da, which is in the range of NF.

**Table 1:** Pure water contact angles, surface charge and MWCO of the membranes.

Membranes	Contact angle (°)	Surface charge, mC/m^2^ (at pH 7.0)	PEG (1500 Da) rejection %
0-M1	77.3	−7.58	88.77
0.05-M2	65.3	−10.78	89.09
0.1-M3	58.4	−12.21	90.33
0.2-M4	53.1	−13.34	90.86
0.3-M5	51.1	−13.39	92.11

### Surface morphology

**Figure 2:**
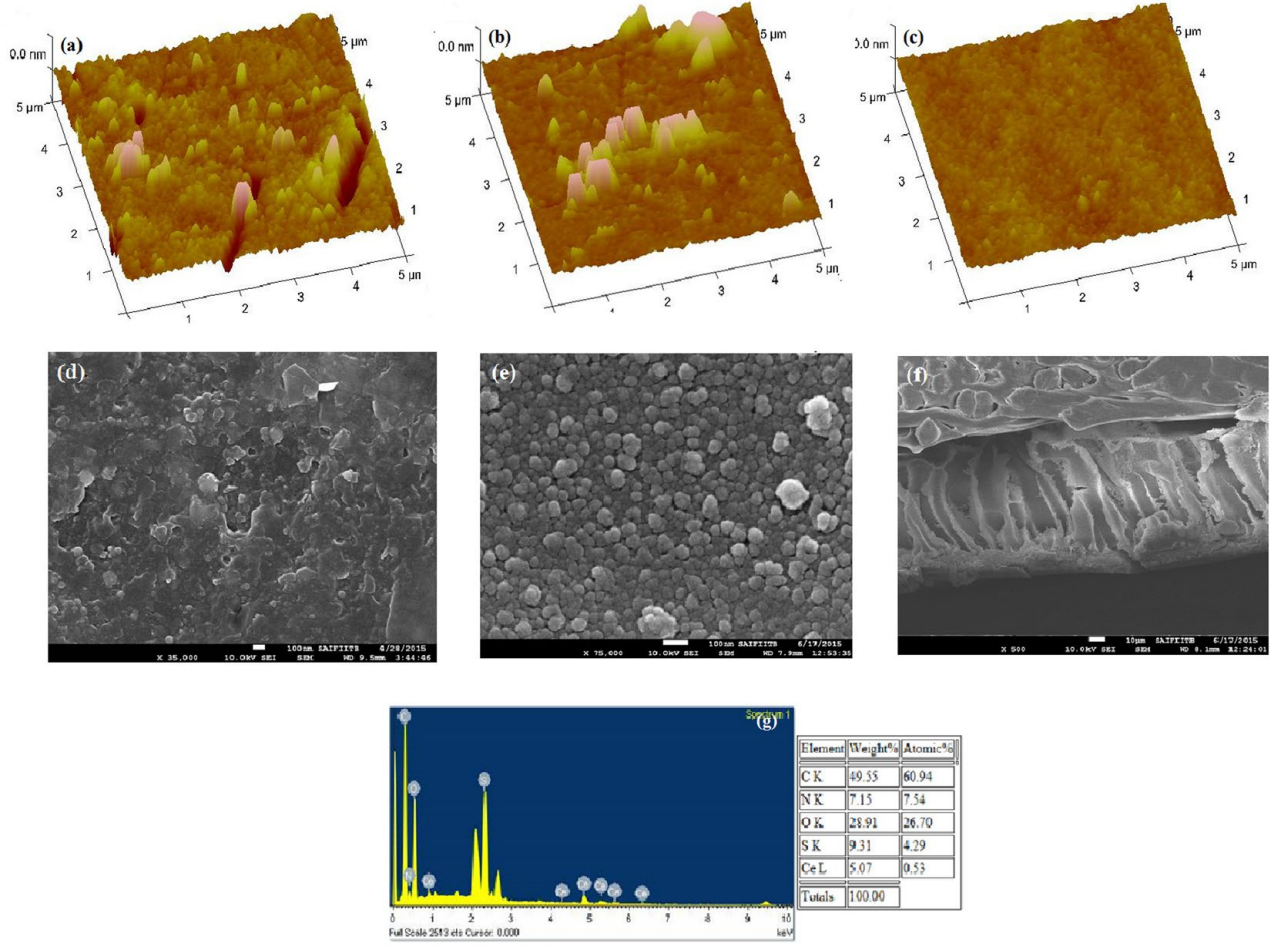
3D SPM and SEM morphology of membranes: SPM images (**a**) 0-M1, (**b**) 0.05-M2 and (**c**) 0.2-M4 and SEM images (**d**) 0-M1 (**e**) 0.2-M4 (**f**) 0.2-M4 cross-section image and (**g**) 0.2-M4 EDS analysis.

The surface roughness and morphologies of the prepared CeO_2_-PA TFN membrane are analyzed by contact mode SPM and SEM (Fig. 2) analysis.

SPM analysis quantifies the surface quality (either smooth or rough) of the membrane. This is an important estimation for the membrane’s anti-fouling study. A membrane with low roughness has possessed a stronger anti-fouling and anti-bio-fouling activity. Here, the CeO_2_ NPs are embedded in the PA layer that changes the surface roughness. The surface roughness of membrane decreases with increasing the concentration of CeO_2_  NPs. Increase in CeO_2_ NPs concentration reveals proper dispersion and distribution of NPs with the thin covering of TMC monomer layer on the surface. This tends to have increased the smoothness of the surface compared to control membrane. The roughness of PA membrane is 8.48 nm (0-M1, Fig. 2a). After deposition of CeO_2_ NPs, the CeO_2_-PA TFN membrane surface becomes smoother with 7.28 nm (0.05-M2, Fig. 2b), 4.79 nm (0.1-M3) and 2.02 nm roughness (0.2-M4, Fig. 2c), because the surface is totally covered by the proper dispersion and distribution of CeO_2_ NPs embedded PA layer. This type of nanopattern arranged surface (smoother surface) based on PA layer removes the foulants from the surface by simply rinsing with water.

SEM analysis observes the morphology of the membrane surface. Fig. 2e shows the seed-like surface morphology by embedding CeO_2_ NPs compared with flaky structure (Fig. 2d) visible on the surface of PA layer coated membrane (0-M1). SEM (0.2-M4, Fig. 2e), reveals reasonably good dispersion of CeO_2_ NPs that are attributed to the covering of thin layer on the surface of CeO_2_ NPs (Fig. 2e). This covering provides the stable attachment of CeO_2_ NPs on the CeO_2_-PA TFN membrane surface, which is confirmed by the cross-sectional view (Fig. 2f), and its cross-sectional EDS analysis (Fig. 2g). The TEM analysis of PA membrane (0-M1) and CeO_2_ NPs embedded (0.2-M4) membrane, shown in the Fig. S3.

### Surface composition

The surface chemical vibrations of the prepared membrane are observed by the ATR-FTIR spectroscopy (Fig. S4). The peak at 1665 cm^−1^ can be credited to the amide I band, C–N and C=O stretching vibrations. A peak at 1608 cm^−1^ is due to aromatic amide [C C ring] breathing as well as N–H deformation vibration and 1243 cm^−1^ for C–N peak of the amide group. The intensity of C=O and C–N stretching is increased with increasing the concentration of CeO_2_ NPs (Fig. 1). The peak at 1541 cm^−1^ can be credited to N–C stretching and N–H bending of –CO–NH– group.

### Mechanical properties

The mechanical strength and elongation properties of modified membranes are shown in Table 2. The tensile strength of the PES support membrane is 5.0 MPa. This is closer to the results of other researchers, where the value is 5.3 MPa. Table 2 represents the effect of the content of CeO_2_ NPs on mechanical properties of the modified nanocomposite membrane. The elongation is 14.18% and tensile strength of membrane is 45.2 MPa when the optimal content of CeO_2_ NPs 0.2 (0.2-M4) in the membrane. The results show that mechanical properties of the membrane are improved with the addition of CeO_2_ NPs. The IP zone is formed by the cross-linked network between MPD, CeO_2_ NPs, and TMC. As the amount of CeO_2_ NPs more (0.3-M5), the mechanical stability of the membrane is getting damaged and membrane becomes weakened. The prepared membrane 0.2-M4 with an appropriate amount of CeO_2_ NPs improved the membrane’s mechanical strength.

**Table 2:** Mechanical properties of the membranes.

Membranes	Mechanical properties	
	Tensile strength (MPa)	Elongation (%)
PES support	5.0	0.04
0-M1	13.2	4.31
0.05-M2	25.6	7.42
0.1-M3	30.8	10.33
0.2-M4	45.2	14.18
0.3-M5	39.3	13.68

### Anti-bacterial and anti-fouling study

**Figure 3:**
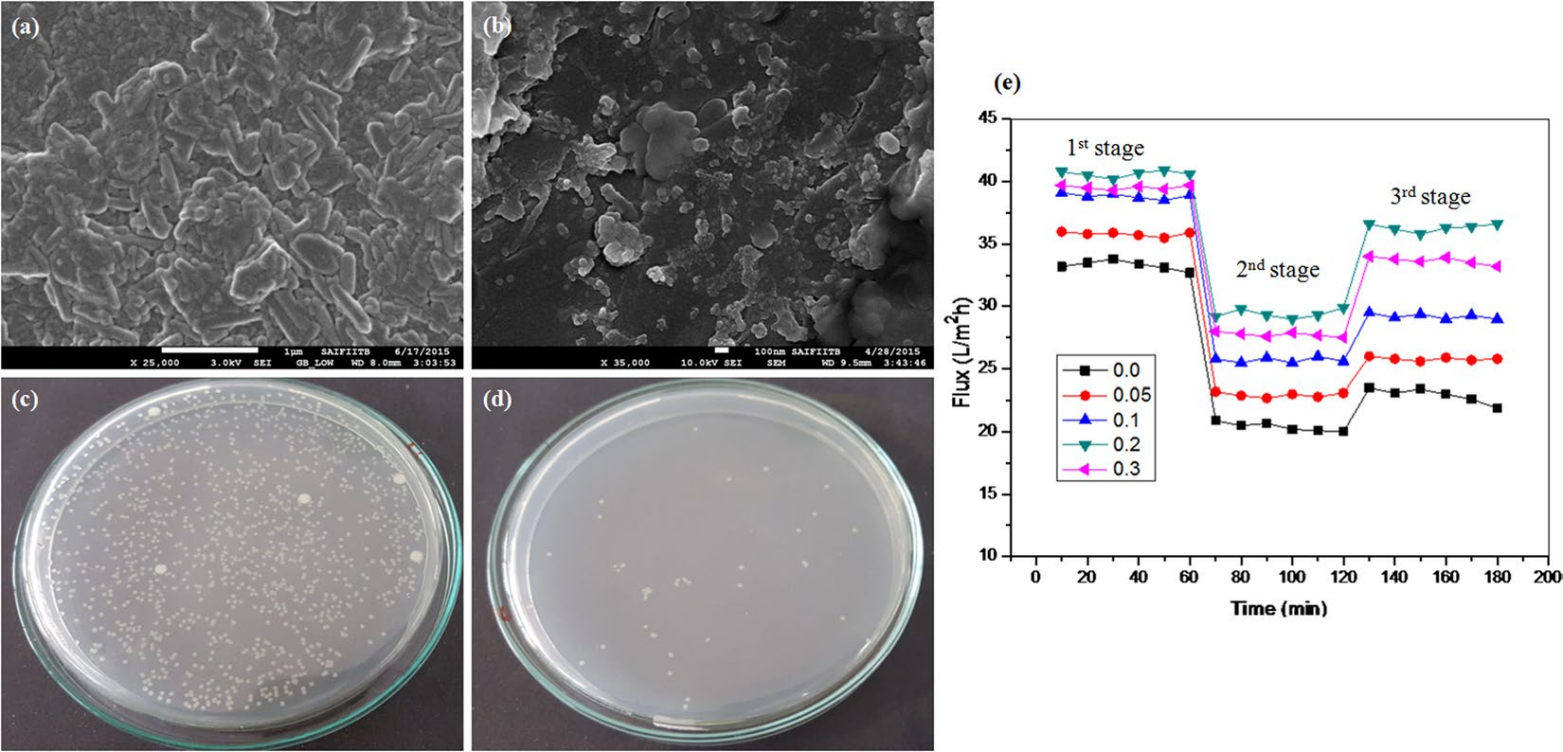
Antibacterial activity by SEM analysis: (**a**) *E. coli* bacteria attached surface morphology of PA membrane and (**b**) CeO_2_ NPs embedded membranes (incubation for 12 h); digital images observing *E. coli* bacterial culture plates: (**c**) 0-M1 PA (**d**) CeO_2_ embedded membrane; Flux recovery experiment: (**e**) permeate flux recovery by saltwater (Suvali beach, Surat, India): 1^st^ stage (pure water), 2^nd^ stage (saltwater) and 3^rd^ stage (again pure water).

The anti-bacterial and anti-fouling activity of prepared CeO_2_-PA TFN membranes is analyzed by using *E. coli* bacteria and saltwater (seawater) (for the composition of saltwater see Table S2), shown in Fig. 3. More fouling resistant, lesser the attachment of foulants and the membrane surface is cleaner.

Anti-bacterial activity of the prepared membrane was analyzed by SEM analysis (Fig. 3a,b) to observe the presence of bacterial cell on the membrane surface. For this analysis membrane was dipped into 5 ml inoculated 10^6^ CFU per ml of *E. coli* solution, which is incubated at room temperature. After that membrane was removed from the solution, dried it and take the surface morphology. It shows that the PA membrane (0-M1) is fully covered with the rod-shaped bacterial cell (Fig. 3a), indicating that the membrane has no antibacterial activity compared to CeO_2_ NPs embedded membrane (0.2-M4), shows the large damage portion of bacterial cells (Fig. 3b) on the surface. The mechanisms can be explained by the fact that the CeO_2_ NPs is attached to the bacterial cell, causing functional and structural damage and inhibits its growth, leading to the cell death. Anti-bacterial activity is further confirmed by observing the presence of viable cells on Petri plates (Fig. 3c,d). For observing the presence of viable cells, the membrane is dipped into *E. coli* solution, which is also incubated at room temperature. After that, it is moved from the solution, rinsed with normal saline water. The rinse solution is further diluted with distilled water till the concentration became 10^−3^ of the original. Then this diluted solution (0.1 ml) is spread onto the nutrient agar (NA) culture and incubated at 37 °C to observe the viable cells. This result shows that the solution treated with the CeO_2_ embedded membrane (0.2-M4), shows significantly decreased in a number of colonies (Fig. 3d) as compared with the PA membrane (0-M1) (Fig. 3c). Considering the reasonably low leaching rate (Table S6) at the steady state, the membrane is expected to be effective for long-term for its antibacterial properties due to the strong bonding between the CeO_2_ NPs and PA thin layer.

Anti-fouling performance is further studied by observing the flux recovery experiment (Fig. 3e) using cross-flow membrane filtration setup ( Fig. S5). In Fig. 3e, 1^st^ and 3^rd^ stages are of pure water (0–60 min and 120–180 min) and 2^nd^ stage is of saltwater (60–120 min). In the 1^st^ step, the pure water flux of the CeO_2_ NPs embedded CeO_2_-PA TFN membranes (0.05-M2, 0.1-M3, 0.2-M4 and 0.3-M5) is greater than that of the PA membrane (0-M1). The abrupt decline of flux in the 2^nd^ stage is likely due to the increased in osmotic pressure of the saltwater. During the 2^nd^ stage, the PA membrane (0-M1) gets more fouled than in the previous stage. 3^rd^ stage is the recovery of pure water flux, and CeO_2_-PA TFN membrane (0.2-M4) shows two times higher flux recovery due to the lower fouling. Moreover, the rejection performance of saltwater is also observed on the basis of conductivity, and the results are shown as Fig. S6. Table 3 represents flux recovery ratio (FRR) and flux reduction (FR). It shows that the FRR of 0.2-M4 membrane is higher (86.94%), while 0-M1 membrane shows lesser (66.59%) recovery. Here, high flux recovery of the membrane (0.2-M4) can be credited to its higher hydrophilicity (53.1°), and high surface negativity (−13.34 mC/m^2^). An improvement in the hydrophilicity and surface negativity comes out to be an acceptable approach to improve self-cleaning ability to overcome membrane fouling problem. Natural foulants (hydrophobic substance) present in the saltwater has been described as negatively charged substance^23^ that is preferred to attach to the positively charged substances. The charge of the natural foulants present in the saltwater (−7.68 mV) is measured using zeta potential measurement.

**Table 3:** Fouling resistance of all the modified membranes.

Membranes	Fouling resistant (%)	
	FRR	FR
0-M1	66.59	38.66
0.05-M2	72.06	36.03
0.1-M3	75.48	33.78
0.2-M4	86.94	27.57
0.3-M5	85.3	29.74

### Salt rejection performance

**Figure 4:**
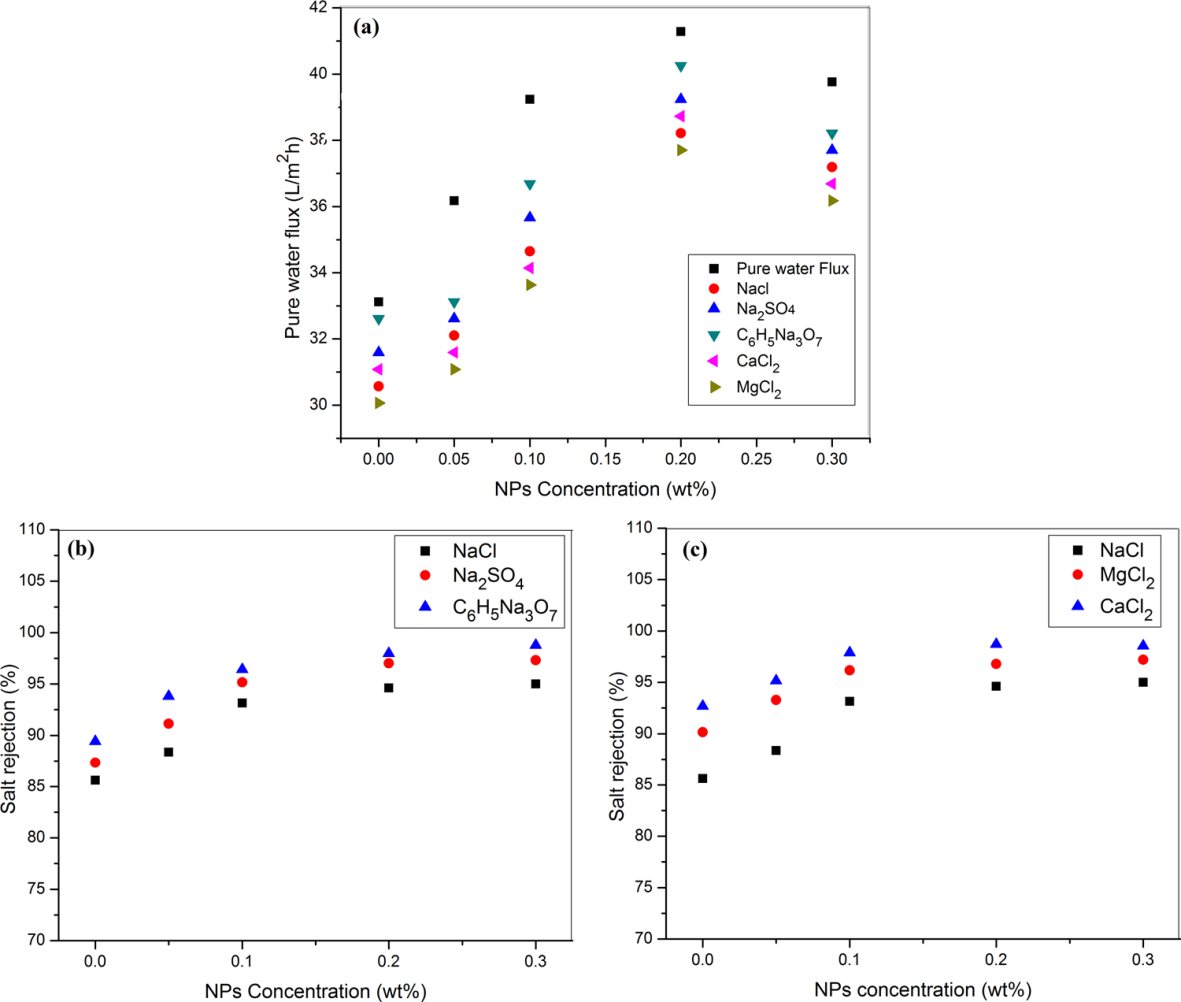
(**a**) Flux of pure and different salt water, salt rejection: (**b**) NaCl, Na_2_SO_4_ and C_6_H_5_Na_3_O_7_, and (**c**) NaCl, CaCl_2_ and MgCl_2_.

The transport phenomena of salts solution through a membrane can be explained by preferential sorption theory. According to this theory, water molecules are sorbs at the membrane interface and then it moves by a viscous flow through the film in which flux is directly proportional to the pressure. Flux and salt rejection performance of the membranes are shown in the Fig. 4.

The pure water flux increases from 33.12 (0-M1) to 41.28 (0.2-M4) L/m^2^h with an increase in the concentration of CeO_2_ NPs up to 0.2 wt% in CeO_2_-PA TFN membranes (Fig. 4a) which show the selectivity of the membrane. The membrane (0.2-M4) prepared with 0.2 wt% of CeO_2_ NPs is showed optimal flux, because it increases up to this point, and then decreases with further increases in the concentration of CeO_2_ NPs (0.3-M5). This decreasing behavior is caused by the aggregation of NPs, which is occurred more easily at the higher concentration, and again disturbed the dispersion and distribution of CeO_2_ NPs into the thin film layer.

The salt rejection follows the order: C_6_H_5_Na_3_O_7_ > Na_2_SO_4_ > NaCl (Fig. 4b) and CaCl_2_ > MgCl_2_ > NaCl (Fig. 4c). Comparison with the available salt rejection data with NF membrane is given in Table S3. The order of salt rejection (Fig. 4b) is predictable from the Donnan characteristic. CeO_2_-PA TFN membrane (0-M1, 0.05-M2, 0.1-M3, 0.2-M4 and 0.3-M5) shows high rejection for low-valence cations and high-valence anions than high-valence cations and low-valence anions (Na_2_SO_4_ > NaCl). It also shows the selectivity of rejection, in which trivalent ions (C_6_H_5_Na_3_O_7_) showed more rejection than the divalent (Na_2_SO_4_) and monovalent (NaCl) ions. Because of the number of cations increases, the size of the molecules increases and forms the bulky molecule which does not pass through the CeO_2_-PA TFN membrane. Na_2_SO_4_ is more rejected than NaCl, which is due to the actions of size sieving processes and electrostatic repulsion. Negatively charged (Table 1) surface containing CeO_2_-PA TFN membranes shows a strong repulsive force against the divalent (SO_4_^−2^) ions than the monovalent (Cl^−^) ions, and prepared CeO_2_-PA TFN membranes are negatively charged under the applied pH of 7. Moreover, the radius of SO_4_^−2^ (3 Å) ions is bigger than that of Cl^−^ (1.95 Å) ions^5^. Therefore, SO_4_^−2^ ions face more resistance than Cl^−^ ions when passing through the CeO_2_-PA TFN membrane. The salt rejection is also prohibited by both the charged surface groups present on the outer layer (Fig. S7) that is the most repulsive under some consideration, i.e. by the negatively charged layer (due to the unreacted acid chloride groups) of the surface for multivalent anions (e.g. C_6_H_5_Na_3_O_7_ and Na_2_SO_4_) and by the positively charged inner layer (due to the unreacted amines) of the thin layer for multivalent cations (CaCl_2_, MgCl_2_, etc.)^39^. Therefore, Ca^2+^ and Mg^2+^ ion (Fig. 4c) has more adsorption ability as compared to Na^+^ ion, because positively charged inner layer and thus follows the sequence of rejection CaCl_2_ > MgCl_2_ > NaCl.

## Conclusions

This work explains the potential of CeO_2_ NPs embedded nanocomposite membrane prepared by pre-seeding interfacial polymerization method for the application of NF process. CeO_2_ NPs is added in the different concentration for the preparation of CeO_2_-PA TFN NF membrane. Analysis results show the CeO_2_ NPs are successfully incorporated in the PA thin layer. CeO_2_-PA (0.2-M4) membrane has the higher flux as compared to PA membrane (0-M1). The surface charged of membrane values shows that the addition of CeO_2_ NPs increases surface hydrophilicity, as well as surface roughness, decreases. These show that the addition of NPs enhances the wettability of nanocomposite membrane. The new type of CeO_2_-PA TFN membranes has the potential to notably improve membrane separation performance and enhancing antibacterial activity against *E. Coli* bacteria. Moreover, as compared to other NPs like silver, CNTs, etc, CeO_2_ NPs have a wide range of applications, so there are so many possibilities of this modified nanocomposite membrane for the desalination study in the future research work.

## Materials and Methods

### Materials

CeO_2_ NPs (~50 nm), sodium lauryl sulfate (SLS), m-phenylenediamine (MPD), trimesoyl chloride (TMC), ethanol and hexane were provided by Sigma-Aldrich, India. Polyethersulfone (PES) porous support (75 kDa) was furnished by Permionics Ltd., Baroda. To evaluate the membranes rejection performance, different salts like sodium chloride (NaCl), sodium sulfate (Na_2_SO_4_), trisodium citrate (C_6_H_5_Na_3_O_7_), calcium chloride (CaCl_2_) and magnesium chloride (MgCl_2_) were purchased by Finar, India. Iodine, barium chloride (BaCl_2_) and potassium iodide (KI) were provided by Fisher Scientific, India. All the chemicals used in this work were analytical grade mark and used without any purification. PEG (Fisher Scientific, India) with different molecular weights were used for MWCO study to analyze the type of membrane. For membrane anti-fouling activity, nutrient broth (NB), nutrient agar (NA) of Himedia, Mumbai, India, and saltwater of Suvali beach, Surat, India were used. The freeze-dried culture of *E. coli* bacteria was collected from MTCC, IMTECH, Chandigarh. The water used in all the experiments was provided by a Milli-Q system (Millipore Elix, India).

### Preparation of CeO_2_ NPs embedded nanocomposite membranes

CeO_2_ NPs embedded nanocomposite (CeO_2_-PA TFN) membranes were prepared on PES porous support by pre-seeding interfacial polymerization method (Fig. S1). In which, first, PES support (diameter: 5.0 cm) was put in an aqueous solution of 0.15 wt% SLS and 2 wt% MPD for 3 min. An excess solution of MPD and SLS were then removed from the surface using tissue paper. A particular amount of CeO_2_ NPs (structure, physical and chemical properties of CeO_2_ NPs are shown in Table S4) (0–0.3 wt %) were dispersed in an organic phase of hexane and 5 wt% ethanol solution, separately, and subjected to sonication for 5 min. Then, 0.05 wt% of trimesoyl chloride was added to this solution and sonicated for 5 min followed by stirring to get well dispersed pre-assembled CeO_2_ NPs. Afterwards, 0.4 ml of this well dispersed pre-assembled sonicated solution was quickly spread on PES support. Spreading was done quickly because of the positive spreading coefficient of hexane solvent. The organic materials that were used to pre-assemble the CeO_2_ NPs on a PES support serve here as seeds. The pre-assembled CeO_2_ NPs have thus got impregnated with the MPD layer to form a stable, defect-free pre-seeded support. This pre-seeded support was then kept in open air at room temperature to evaporate the extra solution. Afterwards, a solution containing 0.1 wt% TMC and hexane was immediately poured onto the pre-seeded PES support and kept for 1 min for polymerization to take place. After the completion of the reaction, membranes were dried in open air at room temperature and then rinsed with distilled water. Five membranes with different concentration of CeO_2_ NPs were prepared (0-M1, 0.05-M2, 0.1-M3, 0.2-M4 and 0.3-M5, wt%) by the same procedure.

### Characterization

Characterization is the first and important step for any prepared/modified membrane to observe its nature, charges, surface chemistry, roughness, pore size, and morphology. A sessile drop manner containing contact angles analysis (Data-Physics OCA15, GmbH, Germany) was used to observe membrane surface nature (hydrophilic or hydrophobic). For that, 1 μL drop of pure water was placed on the dried membrane surface, and the angle was measured with the installed software. The contact angle of buffered and unbuffered electrolytes solution at different pH was also used to calculate the surface charge (using fractional ionization (α), effective acid dissociation constant (pKa) and surface concentration of acidic groups [COO^−^]_o_) of membrane qualitatively by using the Grahame equation^4^ (Table S5). To observe surface chemistry and roughness of the membrane, ATR-FTIR (IRAffinity-1S, Shimadzu) and scanning probe microscopy (SPM, Bruker, USA) analysis were used. Prior to these measurements, the samples were desiccated for 24 h to remove moisture. MWCO study was used to observe the pore size of the membrane using PEG with different molecular weights (600, 1000, 1500 Da). 1000 mg/L solution of PEG was used in the cross-flow filtration module and permeates were collected after attaining the steady-state situation. Comparative molecular weights of PEG in permeate and feed were determined using UV spectrophotometer (DR 6000, HACH, USA) at a wavelength of 535 nm against a reagent blank. Iodine, BaCl_2,_ and KI were used for the sample preparation. Membrane morphologies (surface and cross-section) were analyzed under a FEG-SEM instrument by JSM-7600F (JEOL, Japan). Before imaging, membranes were freeze-dried, cracked in liquid nitrogen, and then covered with gold particles. The morphology of PA membrane and CeO_2_ NPs embedded membrane were investigated using a TEM CM 200 instrument (Philips). The mechanical properties (tensile strength and elongation) of membranes were tested by a tensile testing machine (Tensometer, Kudale PC-2000). The membrane samples were cut into pieces in a particular shape with a total length of 10 cm and width of 1.5 cm and then dried it. The cross head speed was maintaining 20 mm/min.

### Membrane performance

Anti-bio-fouling property of the CeO_2_-PA TFN membrane was analyzed by measuring the growth status of microbial cells on the membrane surface. *E. coli* bacteria were inoculated in the 5 ml of NB solution for 12 h at 37 °C. The membrane (0.03 g) was measured, cut and sterilized for 20 min, respectively. To analyze the antibacterial property, membrane sample was added to 5 ml inoculated 10^6^ CFU per ml of *E. coli* solution, which was incubated at room temperature. After 24 h, the sample was removed from the culture and rinsed with normal saline water. The rinse solution was collected and further diluted with distilled water till the concentration became 10^−3^ of the original. Then the diluted solution (0.1 ml) was spread onto the NA culture and the Petri plates were incubated at 37 °C for 24 h to observe the growth status of microbial cells.

Anti-fouling performance of the CeO_2_-PA TFN membranes was studied by performing flux recovery experiment using saltwater collected from the Suvali beach according to the following steps: (i) pure water flux, (ii) filtration of saltwater solution is performed and flux is measured, (iii) washing with pure water and (iv) again pure water flux is measured. A laboratory-made cross-flow membrane filtration setup was used for all the experiments, in which four stainless steel modules attached in series. The membrane modules were possessed space to put flat-sheet circular membrane part with an area of 18.85 cm^2^. A reciprocating diaphragm type pump (model no.: mROY B-13) was used to pass the feed solution through the membrane. The valve sited at the end of the module was used to pressurize the feed and control the feed pressure. All membranes were initially compressed at 200 psi pressure until steady-state flow was achieved. Flux (J) of pure water and salt solution, different salt rejection (%) experiments, flux recovery ratio (FRR) and flux reduction (FR) of the membranes were carried out at operating pressure 300 psi. Flux, FRR, and FR of the membrane can be calculated using equations (1), (2) and (3) as shown below.

1 $$J=\frac{{V}_{p}}{A\times t}$$

2 $$FRR({\rm{ \% }})=\frac{{J}_{w2}}{{J}_{w1}}\times 100$$

3 $$FR( \% )=(1-\frac{{J}_{f}}{{J}_{w1}})\times 100$$

where, J: the flux (L/m^2^h), A: the membrane area (m^2^), V_p_: the permeate volume (ml) and t: treatment time (h). J_w1_: the average pure water flux during the first stage, J_f_: the feed flux (saltwater) solution and J_w2_: the average of pure water flux during the third stage.

The salt rejection performance of the membranes was observed by filtering NaCl, Na_2_SO_4_, C_6_H_5_Na_3_O_7_, CaCl_2,_ and MgCl_2_ salts solution at an initial concentration of 2000 mg/L. The conductivity of solutions (feed and permeate) was measured by a conductivity meter connected to the system, and a calibration curve was used to relate the solution conductivity to salt concentration. The salt rejection, R, was calculated from the equation (4) as shown below:

4 $$R( \% )=(1-\frac{{c}_{p}}{{c}_{f}})\times 100$$

where C_p_: the conductivity of salt in permeates and C_f_: the conductivity of salt in feed solutions.

Leaching Test: The leaching rate of cerium ions from CeO_2_ NPs embedded TFN membrane was examined by the cerium stability experiments. So, first CeO_2_ NPs embedded TFN membrane was cut into a particular shape with an area 1 cm^2^ and was subsequently immersed in 20 ml of pure water at room temperature for 2, 4 and 24 h. After determined time frames, the amount of cerium ion in the water was analyzed by UV spectrophotometer.

## Electronic supplementary material

Supplementary Information Cerium Oxide NF Membrane
