# Supplementary material for: Cerium oxide nanoparticles embedded thin-film nanocomposite nanofiltration membrane for water treatment
Source: Sci Rep. 2018 Mar 21;8:4976. doi: 10.1038/s41598-018-23188-7 (PMC5862962; doi:10.1038/s41598-018-23188-7)
Supplement: Supplementary file 1 — Supplementary Information Cerium Oxide NF Membrane [file 41598_2018_23188_MOESM1_ESM.docx]

**SUPPLEMENTARY INFORMATION**

**Cerium oxide nanoparticles embedded thin-film nanocomposite nanofiltration membrane for water treatment**

**Sonia R. Lakhotia^ab^, Mausumi Mukhopadhyay ^b *^, Premlata Kumari^a^**

^a^Applied Chemistry Department, Sardar Vallabhbhai National Institute of Technology,

Surat-395007, Gujarat, India.

^b^Department of Chemical Engineering, Sardar Vallabhbhai National Institute of Technology, Surat-395007, Gujarat, India.


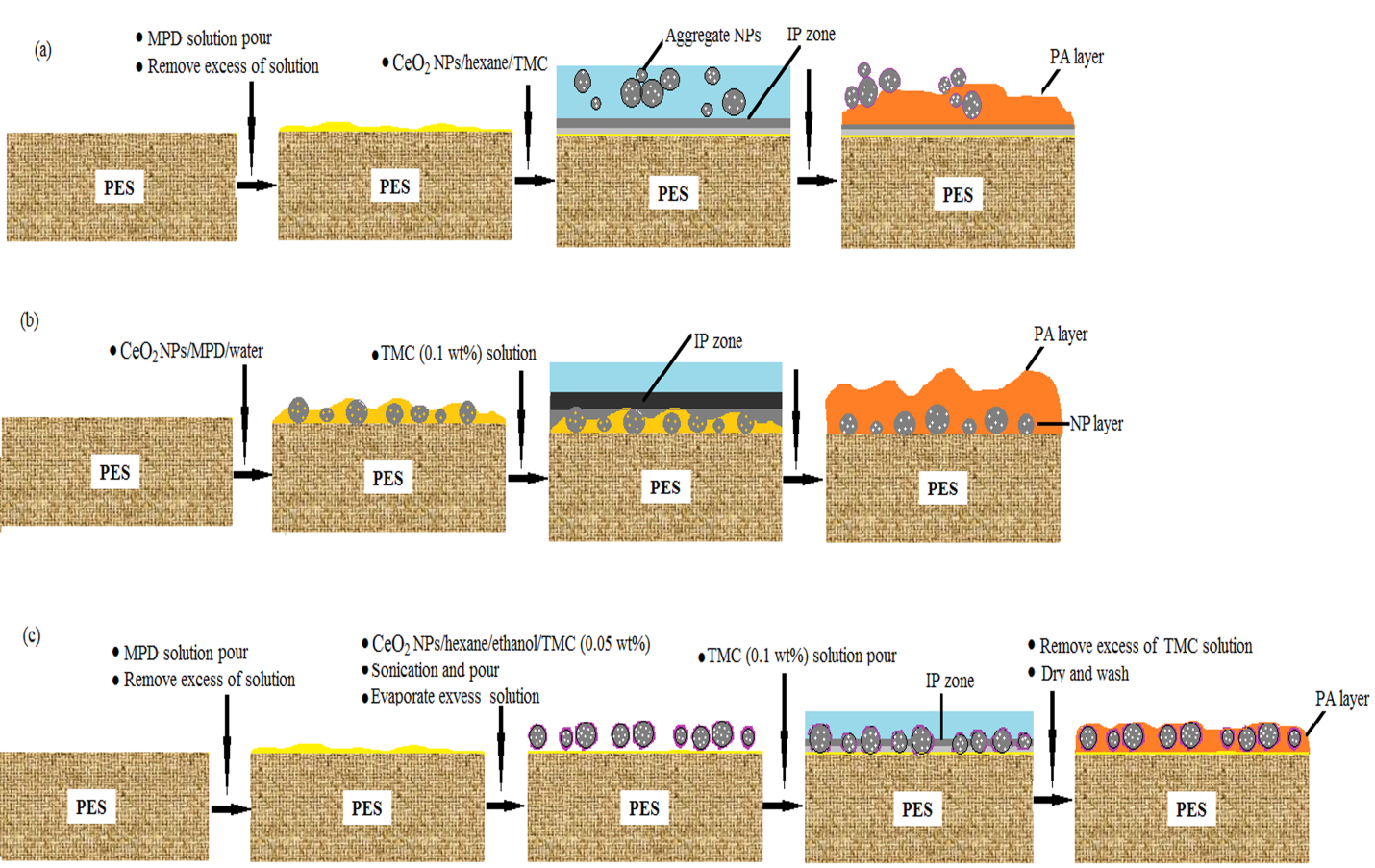


**Supplementary Figure S1.**

(a) Conventional method can be done in two ways: (i) by dispersing NPs in TMC solution (ii) by dispersing NPs in MPD solution and (b) pre-seeding polymerization method.


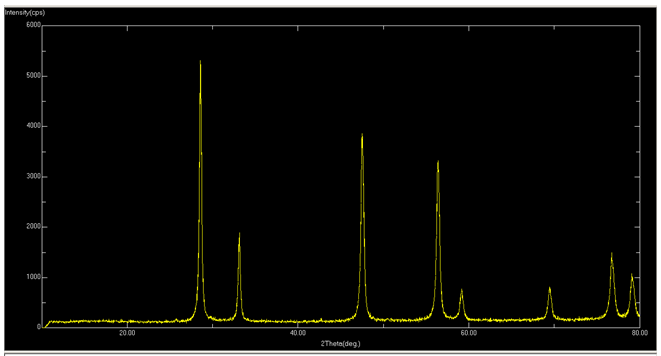


**Supplementary Figure S2.**

XRD analysis of commercial available CeO_2_ nanoparticle.


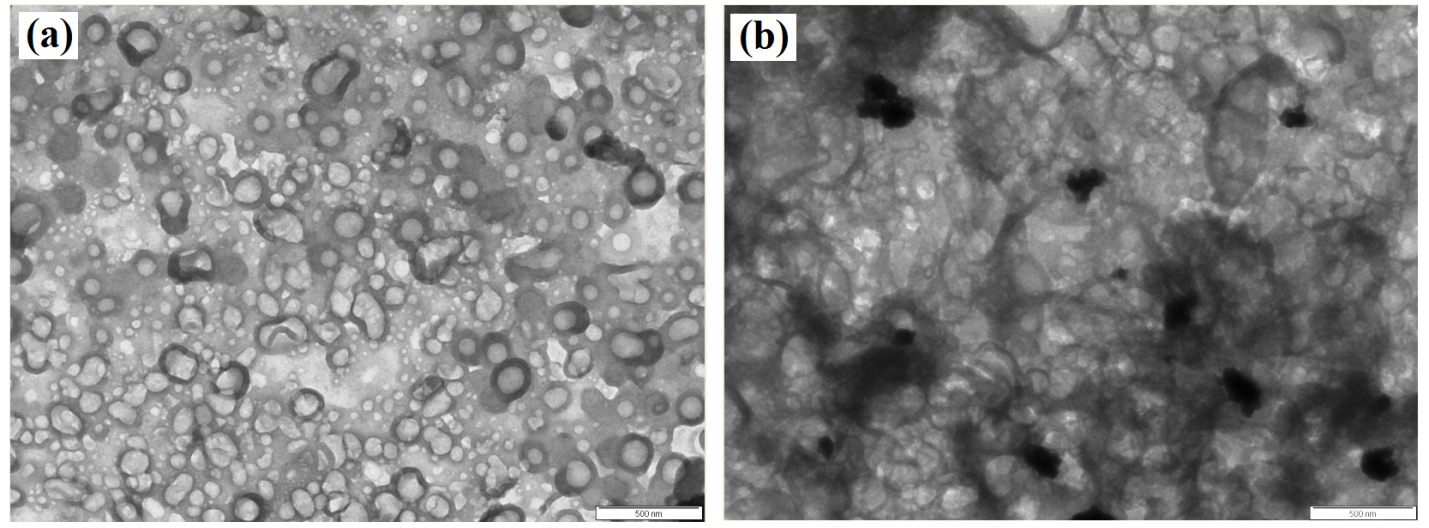


**Supplementary Figure S3.**

TEM Image of membranes: (a) 0-M1 polyamide membrane and (b) 0.2-M4 CeO_2_ NPs embedded membrane.

**
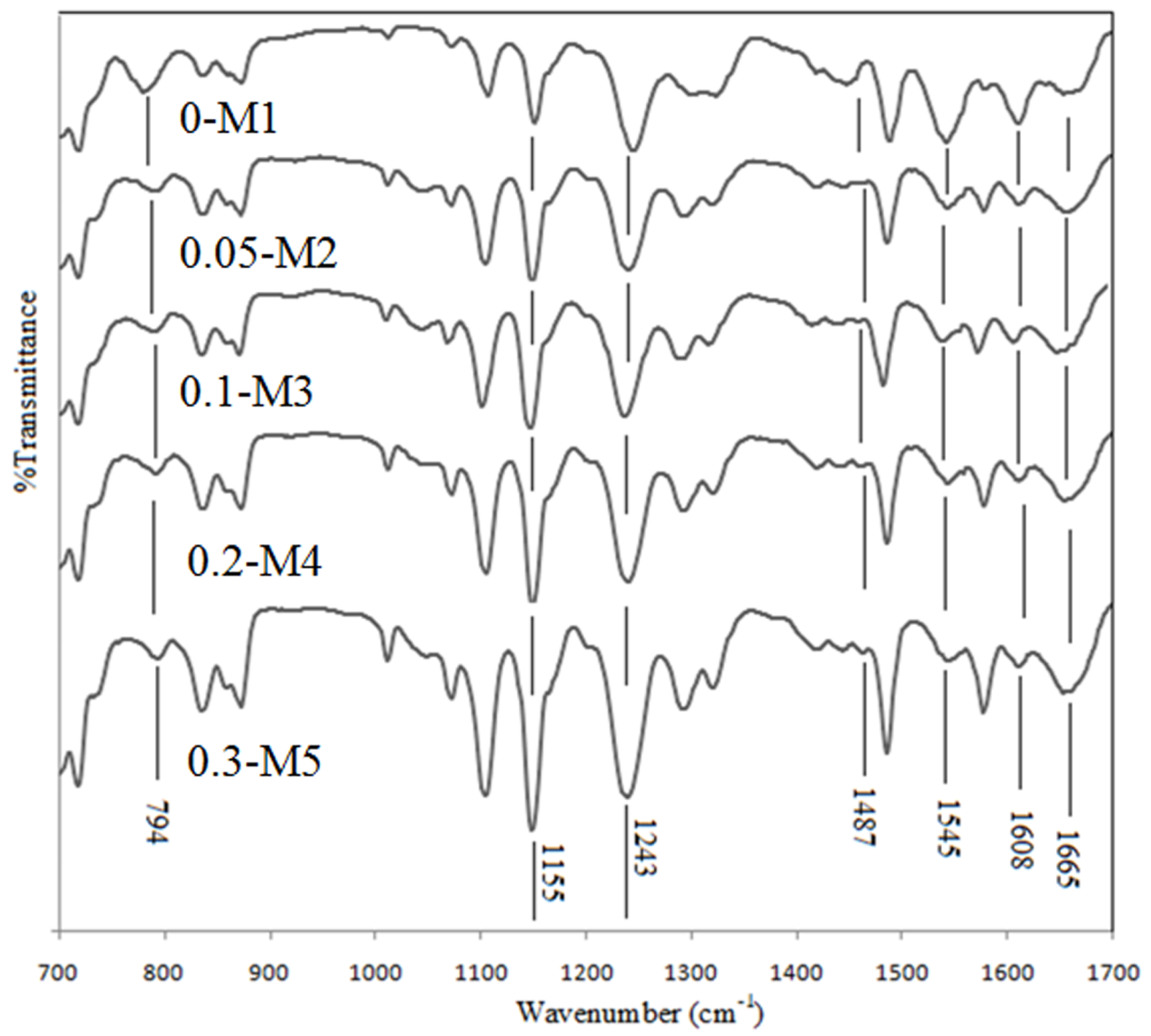
**

**Supplementary Figure S4.**

ATR-FTIR spectra of CeO_2_ NPs embedded TFN NF membrane.

**
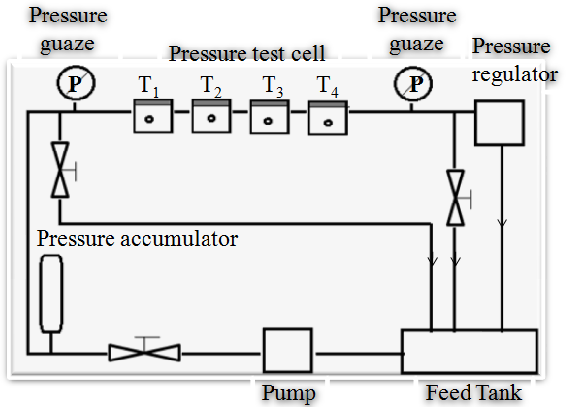
**

**Supplementary Figure S5.**

Schematic diagram of cross-flow membrane filtration system.


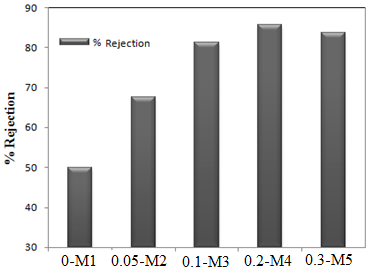


**Supplementary Figure S6**

Rejection performance of membranes using saltwater (Suvali beach, Surat, India) on the basis of conductivity measurements.

**
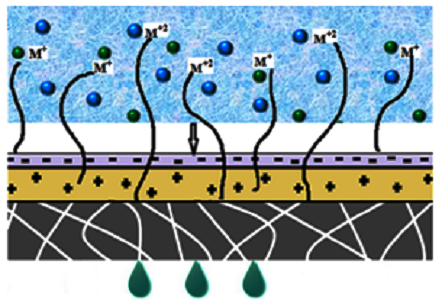
**

**Supplementary Figure S7.**

Schematic diagram of mechanism of salt rejection of CeO_2_-PA TFN membrane.

**Supplementary Table S1.** Rejection performance of prepared membrane using PEG (600, 1000, 1500 Da) solution.

| **Membrane** | **Initial abs.** | **Initial conc.** | **Final abs.** | **Final conc.** | **R %** |
| --- | --- | --- | --- | --- | --- |
| **PEG 600** | | | | | |
| 0-M1 | 2.242 | 1027.37 | 1.714 | 555.55 | 45.92 |
| 0.05-M2 | 2.242 | 1027.37 | 1.654 | 510.61 | 50.29 |
| 0.1-M3 | 2.242 | 1027.37 | 1.562 | 445.13 | 56.67 |
| 0.2-M4 | 2.242 | 1027.37 | 1.499 | 402.68 | 60.82 |
| 0.3-M5 | 2.242 | 1027.37 | 1.488 | 395.47 | 61.50 |
| **PEG 1000** | | | | | |
| 0-M1 | 2.537 | 1039.68 | 1.778 | 396.47 | 61.86 |
| 0.05-M2 | 2.537 | 1039.68 | 1.754 | 380.71 | 63.38 |
| 0.1-M3 | 2.537 | 1039.68 | 1.653 | 317.45 | 69.46 |
| 0.2-M4 | 2.537 | 1039.68 | 1.592 | 281.66 | 72.90 |
| 0.3-M5 | 2.537 | 1039.68 | 1.532 | 248.22 | 76.12 |
| **PEG 1500** | | | | | |
| 0-M1 | 2.894 | 1054.85 | 1.581 | 125.91 | 89.06 |
| 0.05-M2 | 2.894 | 1054.85 | 1.532 | 105.69 | 89.98 |
| 0.1-M3 | 2.894 | 1054.85 | 1.522 | 101.69 | 90.35 |
| 0.2-M4 | 2.894 | 1054.85 | 1.511 | 97.34 | 90.77 |
| 0.3-M5 | 2.894 | 1054.85 | 1.467 | 80.47 | 92.37 |

**Supplementary Table S2.** Composition of saltwater (Suvali beach, Surat, India).

| Parameters | Concentration |
| --- | --- |
| Conductivity, mS/cm | 48.5 |
| Turbidity, NTU | 0.4 |
| Total dissolved solids (TDS), mg/L | 36,860 |
| Total suspended solids (TSS), mg/L | 1.3 |
| Salinity, ppt | 50.79 |
| pH | 8.2 |
| Sodium, mg/L | 10,296 |
| Calcium, mg/L | 560 |
| Magnesium, mg/L | 1,068 |
| Potassium, mg/L | 376 |
| Chloride, mg/L | 28,116 |
| Fluoride, mg/L | 1.2 |

**Supplementary Table S3.** Comparative study of surface modified NF membrane.

| **Type of NF membrane** | **MWCO (Da)** | **Concentration** | **Rejection (%)** | **Ref.** |
| --- | --- | --- | --- | --- |
| NTR7450 (Nitto Denko) | 600-800 | 100 eq.m^-3^, 1-260 gL^-1^ NaCl, 2000 mgL^-1^ MgSO_4_ | NaCl- 41to50, Na_2_SO_4_-88, and MgSO_4_-56 | [1], [2] |
| CA30 (Hoechst) | 1000 | 100 eq.m^-3^ and 1-260 gL^-1^ NaCl, 2000 mgL^-1^ MgSO_4_ | NaCl-8, Na_2_SO_4_-29, MgCl_2_-20 and MgSO_4_-53 | [1], [2] |
| PES/SPEEK | 1000 | 250, 500 1000 ppm | NaCl- 50 and Na_2_SO_4_-90 | [3] |
| DL6 | 1910 | 500 ppm | Na_2_SO_4_-97 and MgCl_2_-15 | [4] |
| PEI_10%_ | 1040 | 35.5 ppm | NaCl-35 and MgCl_2_-70 | [5] |
| PEI_10%_ | 700 | 250, 500 1000 mgL^-1^ | NaCl-43 and MgCl_2_-75 | [5] |
| CeO_2_-TFN | 1500 | 2000 ppm of C_6_H_5_Na_3_O, Na_2_SO_4_, NaCl, CaCl_2_, MgCl_2_, | greater than 85% (shown in **Figure 4b and 4c**) | Present work |

[1] Schaep, J.; Vandecasteele, C.; Mohammad, A. W.; Bowen, R. Modelling the retention of ionic components for different nanofiltration membranes. Sep. Purif. Technol. 2001, 22−23, 169−179.

[2] Bargeman, G.; Westerink, J. B.; Guerra Miguez, O.; Wessling, M. The effect of NaCl and glucose concentration on retentions for nanofiltration membranes processing concentrated solutions. Sep.Purif. Technol. 2014, 134, 46−57.

[3] Lau, W. J.; Ismail, A. F. Theoretical studies on the morphological and electrical properties of blended PES/SPEEK nanofiltration membranes using different sulfonation degree of SPEEK. J. Membr. Sci. 2009, 334, 30−42.

[4] He, T.; Frank, M.; Mulder, M. H. V.; Strathmann, H.; Wessling, M. Preparation of composite hollow fiber membranes: Co-extrusion of hyydrophilic coatings onto porous hydrophobic support structures. J. Membr. Sci. 2002, 207, 143−156.

[5] Gherasim, C. V.; Luelf, T.; Roth, H.; Wessling, M. Dual charged hollow fiber membranes for low-pressure nanofiltration based on polyelectrolyte complexes: One-step fabrication with tailored functionalities, ACS Appl. Mater. Interfaces 2016, 8, 19145−19157.

**Supplementary Table S4.** The structure, physical and chemical properties of CeO_2_.

| **Cerium (IV) oxide** | Linear with chemical formula CeO_2_ |
| --- | --- |
| Physical state and appearance | Solid |
| Odor | Odorless |
| Taste | Not available |
| Molecular Weight | 172.12 g/mole |
| Color | White. Off-white |
| pH (1% solution/water) | Not applicable |
| Boiling Point | Not available |
| Melting Point | 2600°C (4712°F) |
| Critical Temperature | Not available |
| Specific Gravity | 6.1 (Water = 1) Density: 7.13 |
| Vapor Pressure | Not applicable |
| Vapor Density | 5.9 (Air = 1) |
| Volatility | Not available |
| Solubility | Insoluble in cold water, hot water |
| Ionicity (in Water) | Not available |
| Water/Oil Dist. Coeff. | Not available |

**Supplementary Table S5.** Fractional ionization (α), effective acid dissociation constant (pKa), surface concentration of acidic groups [COO^-^]_o_ and surface charges (σ).

| Membrane | pH | θ | Fractional ionization (α) | Effective acid dissociation constant (pKa) | Surface concentration [COO^-^]_o_ | Surface charges (σ) |
| --- | --- | --- | --- | --- | --- | --- |
| PES | 4 | 82.2 | 0.0416 | 5.3622 | 3.46258E+16 | -0.7214 |
|  | 7 | 75.7 | 0.4020 | 7.1723 | 3.11571E+16 | -6.2720 |
|  | 9 | 70.3 | 0.6956 | 8.6410 | 2.85887E+16 | -9.9570 |
| 0-M1 | 4 | 73.4 | 0.1929 | 4.6213 | 5.9923E+17 | -2.3363 |
|  | 7 | 66.9 | 0.5519 | 6.9094 | 6.80425E+17 | -7.5873 |
|  | 9 | 54.2 | 1.2002 | #NUM! | 5.54291E+17 | -13.440 |
| 0.05-M2 | 4 | 60.4 | 0.1308 | 4.8224 | 7.72229E+17 | -2.6886 |
|  | 7 | 52 | 0.6027 | 6.8189 | 6.72508E+17 | -10.7879 |
|  | 9 | 45 | 0.9572 | 7.6496 | 5.94842E+17 | -15.1548 |
| 0.1-M3 | 4 | 55.3 | 0.0565 | 5.2222 | 1.12632E+18 | -1.5053 |
|  | 7 | 47.4 | 0.5252 | 6.9560 | 9.84305E+17 | -12.2176 |
|  | 9 | 45.7 | 0.6190 | 8.7890 | 9.54818E+17 | -13.9687 |
| 0.2-M4 | 4 | 50.9 | 0.1146 | 4.8875 | 1.65794E+18 | -3.3406 |
|  | 7 | 43.7 | 0.5206 | 6.9641 | 1.45887E+18 | -13.3432 |
|  | 9 | 40.1 | 0.7051 | 8.6212 | 1.36214E+18 | -16.8743 |
| 0.3-M5 | 4 | 49.4 | 0.1065 | 4.9237 | 1.28337E+18 | -2.3959 |
|  | 7 | 37.2 | 0.7487 | 6.5257 | 1.02071E+18 | -13.3967 |
|  | 9 | 33.2 | 0.9260 | 7.9024 | 9.36532E+17 | -15.2024 |

**Method 1.**

Surface charge properties of membranes are most commonly measured through zeta (surface) potentials determined from electro-kinetic measurements. But, electro-kinetic measurement relate the net interaction between a particular electrolyte solution and a charged surface rather than the intrinsic charges of the material, while non-idealities like particular ion adsorption and surface conductance often confuse quantitative understanding of electro-kinetic measurements. One alternative of this measurement is contact angle analysis to calculate the surface charges of interfacial polyamide thin layer coated membrane. A plentiful amount of literature is available on the use of contact angle study for analyzing the interfacial properties of biomaterials and polymers beyond classical estimates of wettability by using the Grahame equation.

The membrane samples were placed on a flat Teflon surface and inserted into a Data-Physics Model-OCA15contact angle analyzer. Droplets of buffered and unbuffered electrolytes were used. Buffered solutions included pH 4, pH 7 and pH 9.2. Additional unbuffered aqueous solutions were prepared by adding 2M CaCl_2_, BaCl_2_, MgCl_2_, or SrCl_2_ to a background concentration of 4M NaCl to obtain a total ionic strength of 10M. Unbuffered aqueous solution pH was adjusted from 2 to 12 by adding NaOH or HCl (ACS Grade, Fisher Scientific) to laboratory deionized water (Milli-Q, Millipore, Germany) or NaCl solutions.

**Quantifying surface charge**

Contact angles of aqueous electrolytes adjusted to different pH values were used to quantify membrane surface charge characteristics. Buffered electrolytes eliminate the influence of surface functional groups on the pH of the drop itself, and therefore, allow the hydroxide ion concentration in solution to directly ionize surface acids (Holmes-Farley et al., 1985). Hence, the buffered contact angle titration curve describes the ionization state of the surface according to:

$$\alpha= \frac{COS\theta-COSA}{COSB-COSA} (1)$$

where α: the fractional ionization, A: upper limit of contact angle and B: lower limit of contact angle of the titration curve.

There are three distinct regions to the ionization curve: (1) upper limit, (2) the transition region, and (3) lower limit. For predominantly acidic surfaces like a polyamide membrane, the upper limit (at acidic pH values) describes the region of surface neutrality in which acidic groups are fully protonated. The lower limit (at basic pH values) represents the region of complete ionization ordeprotonation. The transition region describes fractional ionization states, including the apparent dissociation constant, which occurs at the inflection point (Holmes-Farley et al., 1985; Wenzel et al., 1949). The “acid dissociation constant” (pKa) is derived from the ionization curve using:

$$pKa=pH-log\left( \frac{\alpha}{1-\alpha} \right)\left( 2 \right)$$

Where pKa: attributed to the extent of ionization for a simple monobasic acid, which is a constant and independent of the ionization fraction. However, for more complex chemistries, such as those present at the interface of polyamide membranes containing a mixture of amides, free amines, and free carboxylic acids, the pKa defined by Eq. (2) describes the net ionization. Essentially, the acid dissociation constant for a polybasic acid, increases with ionization because the remaining acidic groups become increasingly difficult to ionize (Holmes-Farley et al., 1985; Wenzel et al., 1949).

Contact angle titrations using unbuffered aqueous electrolytes provide insight into the total charge density of the solid surface. The unbuffered water drop does not maintain a constant pH because it is buffered directly by the surface. The upper limit of the unbuffered titration curve is the pH range where the concentration of surface acidic groups is much larger than the concentration of hydroxide ions in the droplet. The breakpoint of the unbuffered titration curve is the point at which the buffering capacity of the surface acidic groups is exceeded. At this point, the number of hydroxide ions in solution equals the total number of acidic functional groups at the surface, which characterizes the maximum charge density of the membrane (Holmes-Farley et al., 1985). From the unbuffered titration curves, the breakpoint was determined as the pH at which the slope of a sigmoidal fit curve first deviated from zero.

The maximum surface charge density (σ_o)_ was calculated by normalizing the carboxylic acid concentration by the wetted surface area between the liquid drop and the solid surface according to:

$$The surface charge \sigma=\sigma_{o}\alpha(3)$$

$$\sigma_{o}= \frac{-e\left[ \mathrm{COO}^{-} \right]_{o}}{r} (4)$$

$$\left[ \mathrm{COO}^{-} \right]_{o}= \frac{{10}^{-\left( 14-\mathrm{pH}_{\mathrm{bp}} \right)}\left( V \right)N_{\mathrm{AV}}}{\left( \pi^{1/3}V^{2/3}\sin^{2}\theta\right)\left( \frac{2}{3}-cos\theta+\left( \cos^{3}\theta/3 \right) \right)^{-2/3}} (5)$$

Here pH_bp_ is the pH at which the titration curve breaks off from horizontal, N_Av_ is Avogadro’s number, e is the electronic charge, [COO^−^]_o_ is the surface concentration of acidic groups, and V is the volume of the liquid drop.

**MWCO study for the pore size of the membrane**

MWCO measurements were determined using polyethylene glycol (PEG) solutes. PEG (Fisher Scientific, India) with molecular weights (600, 1000, 1500 Da) was mixed in pure water. 1000 mg/L solution of PEG was used in the cross-flow membrane filtration setup. Each PEG solution was circulated for about 1 h until the whole system reached steady state, then the permeate was collected for a predetermined period and the volume was measured before being analyzed. Two to three samples were taken for analysis. The modules were thoroughly flushed with deionized water between runs of different molecular weight solutes. It was found the pure water permeation flux in membrane module washing between solute changes did not change, indicating no fouling occurred in the tests. Comparative molecular weights of PEG in permeate and feed were determined using UV spectrophotometer (DR 6000, HACH, USA). Iodine (Fisher Scientific, India), barium chloride (Fisher Scientific, India) and potassium iodide (Fisher Scientific, India) were used for the sample preparation.

Reagents used: 5% (w/v) BaCl_2_ in 1N HCl (100 ml)

2% (w/v) KI diluted 10 times + 1.27 g I_2_

**Procedure:** Four milliliters of sample solution was added to 1 ml of solution prepared by dissolving 1.27g I_2_ in 100 ml 2% KI (w/v) solution was added which is further diluted 10 times. Green color was allowed to develop for 15 min at room temperature, and absorption was read using a spectrophotometer at 535 nm against a reagent blank.

The solute separation factor was calculated by using the following equation:

$$R= \left( 1-\frac{C_{p}}{C_{f}} \right) \times100 (7)$$

where *C*_p_ and *C*_f_ are the solute concentration in the permeate and in the feed solution, respectively.

The solute rejection data were further used to estimate the pore size of the membranes. It is defined as the lowest molecular weights at which greater than 90% of solute with a known molecular weight is retained by the membrane.

**Supplementary Table S6. Stability of CeO_2_ NPs-containing nanocomposite membranes.**

| Membranes | Concentration of CeO_2_ NPs after 2h (ppm) | Concentration of CeO_2_ NPs after 4h  (ppm) | Concentration of CeO_2_ NPs after 24h  (ppm) |
| --- | --- | --- | --- |
| 0-M1 | - | - | - |
| 0.05-M2 | - | 0.04 x 10^-3^ | 0.04 x 10^-3^ |
| 0.1-M3 | - | 0.12 x 10^-3^ | 0.12 x 10^-3^ |
| 0.2-M4 | - | 0.04 x 10^-3^ | 0.04 x 10^-3^ |
| 0.3-M5 | - | 0.08 x 10^-3^ | 0.08 x 10^-3^ |
